# Oral mosapride can provide additional anti-emetic efficacy following total joint arthroplasty under general anesthesia: a randomized, double-blinded clinical trial

**DOI:** 10.1186/s12871-020-01214-4

**Published:** 2020-12-03

**Authors:** Jinwei Xie, Yingchun Cai, Jun Ma, Qiang Huang, Fuxing Pei

**Affiliations:** 1 Department of Orthopaedic Surgery, National Clinical Research Center for Geriatrics, West China Hospital, Sichuan University, No. 37 Guoxue Road, Chengdu, Sichuan Province 610041 People’s Republic of China; 2 Department of Orthopaedic Surgery, The First Affiliated Hospital of Zhengzhou University, No. 1 East Jianshe Road, Zhengzhou, 450052 People’s Republic of China

**Keywords:** PONV, Total joint arthroplasty, General anesthesia, Bowel function

## Abstract

**Background:**

We sought to determine (1) whether the addition of prophylactic oral mosapride to a protocol including dexamethasone and ondansetron further reduces postoperative nausea and vomiting (PONV) compared with ondansetron alone or the combination of both; (2) whether preemptive application of oral mosapride provides additional clinical benefits for bowel function and appetite, thus improving functional recovery.

**Methods:**

We randomized 240 patients undergoing total hip and knee arthroplasty to receive placebo (Control, *n* = 80), dexamethasone (10 mg) before anesthesia induction (Dexa, *n* = 82), or dexamethasone (10 mg) before anesthesia induction as well as oral mosapride (5 mg) before and after surgery (Mosa+Dexa, *n* = 78). Patients were assessed at 0–6, 6–12, 12–24, and 24–48 h postoperatively. Primary outcomes were incidence and severity of PONV as well as complete response. Secondary outcomes were appetite, time until first defecation and ambulation, patient satisfaction score, and length of hospital stay.

**Results:**

Mosa+Dexa patients showed significantly lower incidence of nausea at 6–12 h (3.8%) and over the entire evaluation period (6.4%), as well as a higher rate of complete response (89.7%) than other patients. Mosa+Dexa patients required less time to achieve first defecation and ambulation, they were hospitalized for shorter time, and they were more satisfied with clinical care.

**Conclusion:**

Addition of oral mosapride further reduced incidence of PONV, especially postoperative nausea, during 6–12 h postoperatively. Moreover, preemptive application of oral mosapride can further improve appetite, bowel function, ambulation and length of hospital stay.

**Trial registration:**

The study protocol was registered at the Chinese Clinical Trial Registry (ChiCTR1800015896), prospectively registered on 27/04/2018.

## Background

Total hip and knee arthroplasty remain one of the most commonly performed major orthopedic procedures; the number of procedures in most countries has increased rapidly over the past decades [1, 2]. Postoperative nausea and vomiting (PONV) are one of the most common and distressing complications after surgery, especially when the surgery is performed under general anesthesia: PONV occurs in 25–30% of all patients, and the rate can reach 80% among at-risk patients without prophylactic intervention [3, 4]. PONV can lead to dehydration, hypertension and other postoperative morbidities, which may prolong hospital stay and increase risk of readmission, raising healthcare costs [5]. This was a paradox in the circumstance that surgeons and healthcare providers have been shifting their focus from the surgical technique to perioperative management in order to improve patients’ psychological and functional recovery [6].

Several prophylactic interventions have been reported to prevent and treat PONV, e.g. 5-HT_3_ receptor antagonists, NK-1 receptor antagonists, corticosteroids, butyrophenone and antihistamines [7]. While these measures can be effective, PONV remains a persistent problem [8, 9]. One reason for this persistence is the gap between implementation and our goal of a “PONV-free hospital”. We previously found that PONV occurred in up to 48.8% of patients undergoing total joint arthroplasty under general anesthesia at our medical center (unpublished data). Another reason for this persistence is that anti-PONV measures can be associated with adverse effects. For example, use of the 5-HT_3_ receptor antagonist ondansetron can aggravate the postoperative constipation that occurs in up to 65% of total joint arthroplasty patients [10]. We have shown that low-dose dexamethasone can reduce the incidence of PONV following total hip and knee arthroplasty [11, 12], but it can be contraindicated in the presence of diabetes or gastrointestinal ulcers. Therefore, it’s necessary and practical to search for other antiemetic protocol.

The major risk factor of PONV is use of opioids, which can stimulate the release of 5-HT and inhibit gastrointestinal peristalsis [13]]. Indeed, selective 5-HT_4_ agonists can stimulate the gastrointestinal tract and promote motility [14]. The selective 5-HT_4_ agonist mosapride can reduce vomiting caused by chemotherapy [15]. This inspired us to examine whether the anti-emetic effects of oral mosapride might offer clinical benefits for total joint arthroplasty patients.

Thus, we sought to determine in the present study (1) whether the addition of prophylactic oral mosapride to a protocol including dexamethasone and ondansetron can further reduce PONV compared with ondansetron alone or the combination of both; and (2) whether preemptive application of oral mosapride can provide additional clinical benefits for recovery of bowel function and appetite.

## Methods

### Study design

This prospective, randomized, clinical trial was performed on patients undergoing primary total hip or knee arthroplasty between November 2017 and December 2018. Institutional review board approval (2012–268) was obtained before the enrollment of patients. All patients provided written informed consent and research authorization before surgery. The study was conducted in compliance with the recommendations of the CONSORT Statement and the Declaration of Helsinki. The study protocol was registered at the Chinese Clinical Trial Registry (ChiCTR1800015896).

### Participants

Eligible patients included those at least 18 years old who were at risk of PONV (at least 1 score of Apfel), and scheduled for primary total hip or knee arthroplasty for end-stage joint diseases such as osteoarthritis, development dysplasia of hip, and osteonecrosis of the femoral head. Exclusion criteria included a history of intolerance of any drugs used in the current study, administration of another anti-emetic drug or systemic steroid within 24 h before surgery, allergy to experimental drugs or history of adverse reactions, diabetes with poor blood glucose control, history of steroid or immunosuppressive drug use within the previous 6 months, history of cardiac disease such as heart failure, heart block, ventricular arrhythmia or severe impairment of bowel motility, renal function or hepatic function.

### Randomization and treatment

Patients in this double-blind study were randomly allocated into three groups using a computer-generated randomization list in a 1: 1: 1 ratio. A random allocation sequence concealed in opaque sealed envelopes only opened before surgery. The control group received 2 ml of normal saline during anesthesia induction, followed by oral placebo at 3 h before surgery and three times per day after surgery. The Dexa group received 10 mg of dexamethasone (in 2 ml) during anesthesia induction, as well as oral placebo at 3 h before surgery and three times per day after surgery. The Mosa+Dexa group received 10 mg of dexamethasone (in 2 ml) during anesthesia induction, as well as 5 mg of oral Mosapride at 3 h before surgery and three times per day after surgery. Dexamethasone was administered intraoperatively by the anesthesiologist and oral drugs were given postoperatively by nurses who were not involved in the study. Patients, surgeons, data collectors and analysts were blinded to group allocation.

### Anesthesia and perioperative pain management

All surgeries were performed under general anesthesia by the same surgeons; standard medial parapatellar arthrotomy was performed for total knee arthroplasty, and the posterolateral approach was used for total hip arthroplasty. A cemented posteriorly stabilized prosthesis was implanted in all total knee arthroplasty patients, and cementless acetabular and femoral components were implanted in total hip arthroplasty patients.

All patients received the same anesthetic regimen and multimodal pain management protocol. Cefuroxime (1.5 g) was given intravenously as prophylactic antibiotic prior to incision. Sufentanil (0.2 μg/kg) propofol (2 mg/kg), atracurium (1 mg/kg) and midazolam (2 mg) were used for anesthesia induction. Then sufentanil (0.1 μg/kg), atracurium (0.5 mg/kg) and sevoflurane (1–3%) were used to maintain anesthesia during surgery. After prosthesis insertion, propofol (4 mg per kg per h) and remifentanil (0.1 μg per kg per min) were used to maintain anesthesia. Anesthetic drugs were discontinued before wound closure. At the end of surgery, 8 mg ondansetron was administered intravenously to all patients.

After prosthesis insertion, a periarticular infiltration of 200 mg ropivacaine (100 mg per 10 ml) in 60 ml of normal saline was injected all around the capsule before closure. A dose of 40 mg of parecoxib was injected intravenously to manage pain. Postoperative pain control consisted of 50 mg of oral voltaren (Diclofenac Sodium Sustained Release Tablets) and 10 mg of oxycodone every 12 h. Breakthrough pain was recorded using a Visual Analgesic Scale (VAS) score that ranged from 0 (no pain) to 10 (most severe). If the VAS score was > 6, 50 mg of pethidine was given as an intramuscular injection when required, up to every 6 h. No nerve block or intravenous patient-controlled analgesia was utilized perioperatively.

### Outcome measurements

The primary outcome variables were the incidence of PONV, severity of PONV and complete response. Secondary outcome variables included time until first defecation and ambulation, postoperative appetite score, patient satisfaction score and length of hospital stay. A blinded clinical investigator reviewed the diagnosis and medical histories of patients and prospectively collected demographic data and surgical information. The investigator also recorded all episodes of nausea and vomiting, severity of nausea, requirement for anti-emetic rescue, and complete response during four postoperative periods (0–6, 6–12, 12–24 and 24–48 h). Nausea was defined as a subjective unpleasant sensation associated with awareness of the urge to vomit, and vomiting was defined as the forceful expulsion of gastric contents from the mouth [16]. The incidence of nausea and vomiting was determined in each of the four periods and during the entire study by calculating the proportion of patients who experienced PONV. In order to avoid double counting, the patients experienced both nausea and vomiting would be counted as episode of vomiting. Following institutional guidelines, 10 mg of intramuscular metoclopramide was used as a first-line anti-emetic rescue treatment when patients experienced two or more episodes of PONV within 2 h. This was followed by 4 mg of intravenous ondansetron when two consecutive boluses of metoclopramide alone, delivered 30 min apart, were ineffective.

We used a standardized scoring algorithm to classify the severity of PONV on a 4-level scale during the 48-h observation period. Complete response was defined as no additional PONV or no requirement for anti-emetic rescue. Mild PONV described the occurrence of mild nausea or one episode of vomiting caused by exogenous stimulus (drinking or movement). Moderate PONV referred to when the patient vomited up to two times or experienced nausea that required anti-emetic rescue only once. Patients were classified as having severe PONV if they suffered more than two emetic episodes or required more than one dose of rescue anti-emetic [17, 18].

The same blinded investigator also recorded the time until first defecation, ambulation and length of hospital stay, and evaluated appetite score on postoperative days 0–2 as follows: 1 point, lower appetite than preoperatively; 2 points, same appetite as preoperatively; and 3 points, more appetite than preoperatively. The same investigator assessed patient satisfaction before discharge using a VAS score that ranged from 0 (extremely dissatisfied) to 10 (very satisfied). During the perioperative period, all the patients would be carefully evaluated for side effects and cardiac complications such as atrioventricular block, or QT interval prolongation with the use of electrocardiogram if necessary.

### Statistical analysis

We performed a priori power analysis based on our preliminary results showing that PONV incidence was 49% in patients receiving ondansetron prophylaxis alone after total joint arthroplasty [unpublished data]. We calculated that 228 patients (76 in each arm) were required to detect a 50% reduction in PONV incidence at an alpha level of 0.05 and a power of 0.9 using a two-sided test. To allow for exclusions and dropouts, we aimed to enroll 246 patients.

Inter-group differences in categorical variables such as incidence of PONV or proportion of complete response were assessed for significance using the chi-square or Fisher’s exact tests. When differences were significant, multiple comparisons between groups were performed using a Bonferroni-corrected post hoc test. Inter-group differences in continuous variables were assessed for significance using either one-way ANOVA, in the case of body mass index, length of hospital stay, time until first defecation or ambulation; or the Wilcoxon signed-rank test, in the case of appetite and patient satisfaction scores. When differences were significant, multiple comparisons between groups were performed using a post hoc Tukey test. Differences associated with *p* <  0.05 were considered statistically significant. All statistical analyses were conducted in SPSS 21.0 (IBM, Chicago, IL, USA).

## Results

**Fig. 1:**
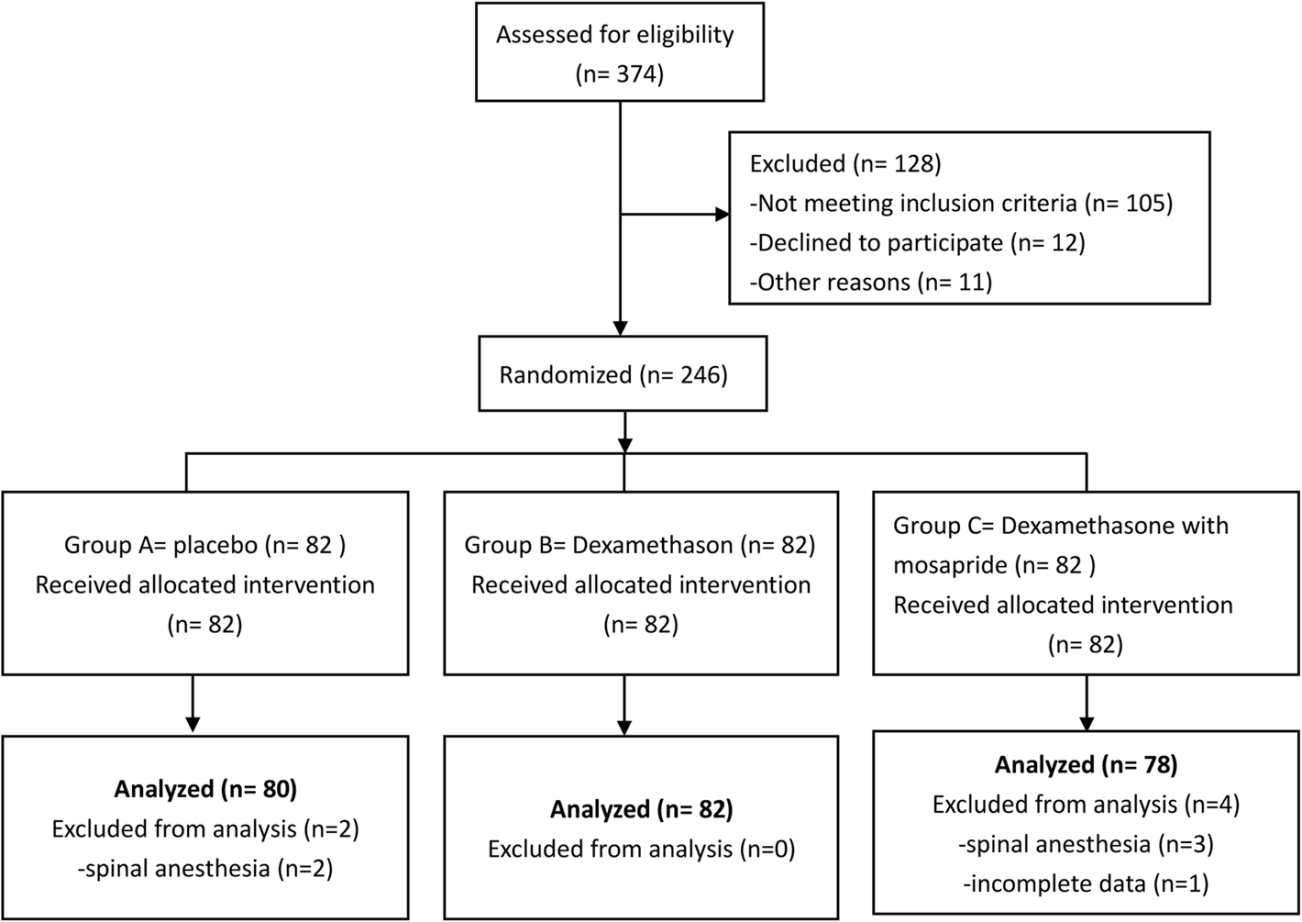
A flow diagram shows the patients recruitment

A total of 82 patients were initially allocated to each of the three groups. We excluded two patients from the control group and four from the Mosa+Dexa group because of incomplete data and spinal anesthesia. In the end, 240 patients (80 in the control group, 82 in the Dexa group and 78 in the Mosa+Dexa group) were included in the final analysis (Fig. 1). These groups showed no significant differences in clinical or demographic characteristics (Table 1).

**Table 1:** Baseline characteristics of all patients

	Control (*n* = 80)	Dexa (n = 82)	Mosa+Dexa (*n* = 78)	p
Age (years)	61.2 (8.9)	62.7 (11.8)	62.2 (12.5)	0.376
Female / Male	47 / 33	61 / 21	53 / 25	0.104
Height (m)	1.61 (0.07)	1.58 (0.07)	1.59 (0.06)	0.055
Weight (kg)	63.4 (13.5)	62.2 (9.0)	60.1 (8.7)	0.143
BMI (kg/m^2^)	24.4 (4.6)	24.9 (3.4)	23.5 (3.0)	0.057
Smoking				0.266
Yes	33 (41.2%)	24 (29.3%)	26 (33.3%)	
No	47 (58.8%)	58 (70.7%)	52 (66.7%)	
History of PONV				0.856
Yes	10 (12.5%)	8 (9.8%)	10 (12.8%)	
No	70 (87.5%)	74 (91.2%)	68 (87.2%)	
History of motion sickness				0.994
Yes	27 (33.8%)	28 (34.1%)	33 (42.3%)	
No	53 (66.2%)	54 (65.9%)	45 (57.7%)	
ASA Score				0.806
2	56 (70%)	55 (67.1%)	56 (71.8%)	
3	24 (30%)	27 (32.9%)	22 (28.2%)	
Surgery				0.439
THA	36 (45%)	31 (37.8%)	37 (47.4%)	
TKA	44 (55%)	51 (62.2%)	41 (52.6%)	
Number of comorbidities				0.978
1	52 (65%)	52 (63.4%)	50 (64.1%)	
≥ 2	28 (35%)	30 (36.6%)	28 (35.9%)	
Operation time (min)	76.3 (21.6)	77.6 (20.1)	76.2 (27.8)	0.920
Anesthesia time (min)	125.5 (26.0)	127.1 (26.5)	120.1 (33.6)	0.280
PACU time (min)	70.3 (48.5)	80.9 (44.7)	76.1 (35.5)	0.300
Sufentanil (μg)	24.24 (2.92)	24.18 (2.59)	24.89 (3.17)	0.195
Remifentanil (mg)	0.58 (0.15)	0.62 (0.10)	0.62 (0.30)	0.329
Propofol (mg)	219.45 (109.40)	196.93 (79.12)	220.54 (178.85)	0.420
Midazolam (mg)	2.09 (0.51)	2.12 (0.41)	2.11 (0.48)	0.946
Atracurium (mg)	13.19 (1.91)	13.37 (2.60)	13.65 (2.18)	0.436
Sevoflurane (ml)	29.97 (6.71)	30.23 (5.47)	30.53 (11.11)	0.913
VAS pain score- rest	3.81 (1.98)	3.58 (1.55)	3.42 (1.26)	0.374

Data presented as mean (stand deviation) or number (percentage)

*ASA* American Society of Anesthesiologists; *BMI* body mass index; *Dexa* dexamethasone; *Mosa* mosapride; *THA* total hip arthroplasty; *TKA* total knee arthroplasty; *PACU*, post-anesthesia care unit

Prophylactic use of concurrent dexamethasone and oral mosapride reduced the overall incidence and severity of PONV, and improved the overall complete response during the entire 48-h evaluation period. Across the entire evaluation period, incidence of postoperative nausea was lowest in the Mosa+Dexa group (6.4%), followed by the Dexa group (19.5%) and the control group (43.8%) (*p* <  0.01 for all, Table 2). Similarly, incidence of postoperative vomiting was lowest in the Mosa+Dexa group (3.9%), followed by the Dexa group (6.1%) and the control group (11.2%), although these differences were not significant (*p* = 0.176). The rate of complete response was significantly higher in the Mosa+Dexa group (89.7%) than in the Dexa group (74.4%, *p* = 0.012) or control group (45%, *p* <  0.001). The rate of severe PONV was significantly lower in the Mosa+Dexa group (1.3%) than in the Dexa group (7.3%, *p* = 0.062) and control group (18.8%, p <  0.001).

**Table 2:** Incidence of PONV during the first postoperative 48 h

	Control (n = 80)	Dexa (*n* = 82)	Mosa + Dexa (*n* = 78)	p*	p1^**†**^	p2^**†**^	p3^**†**^
Total	44 (55%)	21 (25.6%)	8 (10.3%)	< 0.001	**< 0.001**	**< 0.001**	**0.014**
Nausea	35 (43.8%)	16 (19.5%)	5 (6.4%)	< 0.001	**0.001**	**< 0.001**	**0.014**
Vomiting	9 (11.2%)	5 (6.1%)	3 (3.9%)	0.176	NA	NA	NA
Complete response	36 (45%)	61 (74.4%)	70 (89.7%)	< 0.001	**< 0.001**	**< 0.001**	**0.012**
Severe PONV	15 (18.8%)	6 (7.3%)	1 (1.3%)	0.001	0.030	**< 0.001**	0.062

Data presented as number of patients (percentage)

*Dexa* dexamethasone; *Mosa* mosapride; *PONV* postoperative nausea and vomiting

* Uncorrected *p* values (for the three-way comparison)

^**†**^ Bonferroni-corrected *p* values: p1, Control vs Dexa; p2, Control vs. Mosa+Dexa; p3, Dexa vs. Mosa-Dexa. The corrected significance threshold was 0.016

Simultaneous application of mosapride and dexamethasone reduced the incidence of nausea mainly during the first 12 h (Tables 3 and 4): the incidence during 6–12 h was significantly lower in the Mosa+Dexa group (3.8%) than in the Dexa group (15.9%, *p* <  0.016) and control group (32.5%, p <  0.016). Moreover, all vomiting episodes occurred during the first 12 h in the Mosa+Dexa patients, while some vomiting incidents in other groups occurred later (Table 4). Nevertheless, the vomiting incidence during the first 12 h did not differ significantly among the three groups. Less patients in Mosa+ Dexa group (2.6%) required rescue treatment for PONV when compared with Dexa group (9.8%, *p* = 0.06) and Control group (21.3%, *p* <  0.001, Table 5), as well as total dose of metoclopramide. No significances were detected among the three groups regarding the postoperative pain score and rescue requirement.

**Table 3:** Timing of PONV events during the postoperative period

Time	Control (n = 80)	Dexa (n = 82)	Mosa+Dexa (n = 78)	p*	p1^**†**^	p2^**†**^	p3^**†**^
**Nausea**	35 (43.8%)	16 (19.5%)	5 (6.4%)	< 0.001	0.001	< 0.001	0.014
0–6 h	30 (37.5%)	14 (17.1%)	5 (6.4%)	< 0.001	0.003	< 0.001	0.037
6–12 h	26 (32.5%)	13 (15.9%)	3 (3.8%)	< 0.001	0.002	< 0.013	0.011
12–24 h	8 (10%)	3 (3.7%)	0	0.006	0.109	0.007	0.246
24–48 h	6 (7.5%)	1 (1.2%)	0	0.013	0.062	0.028	1.000
**Vomiting**	9 (11.2%)	5 (6.1%)	3 (3.9%)	0.176			
0–6 h	7 (23.8%)	4 (12.2%)	3 (3.8%)	0.380			
6–12 h	3 (12.5%)	2 (6.1%)	1 (1.3%)	0.610			
12–24 h	1 (1.3%)	1 (1.2%)	0	1.000			
24–48 h	1 (1.3%)	0	0	0.652			

Data presented as number of patients (percentage). Dexa, dexamethasone; Mosa, mosapride; PONV, postoperative nausea and vomiting

* Uncorrected values

^**†**^ Bonferroni-corrected p values: p1, Control vs Dexa; p2, Control vs. Mosa+Dexa; p3, Dexa vs. Mosa-Dexa. The corrected significance threshold was 0.016

**Table 4:** Duration of PONV during the first postoperative 48 h

Duration	Control (n = 80)	Dexa (n = 82)	Mosa+Dexa (n = 78)	p*	p1^**†**^	p2^**†**^	p3^**†**^
< 6 h	18 (22.5%)	12 (14.6%)	7 (9.0%)	0.061			
6–12 h	8 (10.0%)	6 (7.3%)	1 (1.3%)	0.068			
12–24 h	8 (10.0%)	2 (2.4%)	0	0.004	0.046	0.007	0.497
> 24 h	10 (12.5%)	1 (1.2%)	0	< 0.001	0.004	0.001	1.000

Data presented as number of patients (percentage)

* Uncorrected values

^**†**^ Bonferroni-corrected p values: p1, Control vs Dexa; p2, Control vs. Mosa+Dexa; p3, Dexa vs. Mosa-Dexa. The corrected significance threshold was 0.016

**Table 5:** The requirement of rescue treatment and postoperative VAS pain score

	Control (n = 80)	Dexa (n = 82)	Mosa+Dexa (n = 78)	p*	p1^**†**^	p2^**†**^	p3^**†**^
Metoclopramide							
Number	17 (21.3%)	8 (9.8%)	2 (2.6%)	0.001	0.043	< 0.001	0.060
Mean dose (mg)	2.88 (40)	1.10 (20)	0.26 (10)	0.002	0.044	0.002	0.496
VAS-rest 24 h	2.66 ± 0.72	2.45 ± 0.91	2.59 ± 0.82	0.333			
VAS-rest 48 h	2.16 ± 0.99	2.03 ± 0.73	2.15 ± 0.71	0.551			
Pethidine							
Number	30 (37.5%)	19 (23.2%)	21 (26.9%)	0.116			
Mean dose (mg)	20 (100)	17.38 (50)	16.99 (100)	0.486			

Data presented as number of patients (percentage), mean (range) or mean ± standard deviation. *Dexa* dexamethasone; *Mosa* mosapride

* Uncorrected p values (for the three-way comparison)

^**†**^ Bonferroni-corrected *p* values: p1, Control vs Dexa; p2, Control vs. Mosa+Dexa; p3, Dexa vs. Mosa-Dexa. The corrected significance threshold was 0.016

The preemptive use of mosapride also reduced the time until first defecation, which was significantly shorter in the Mosa+Dexa group (36.4 ± 18.3 h) than in the Dexa group (54.7 ± 15.8 h, *p* <  0.001) and control group (60.7 ± 24.6 h, p <  0.001; Table 6). Appetite score was significantly higher in the Mosa+Dexa group than in the control group on postoperative days 0 (*p* = 0.019), 1 (*p* <  0.001) and 2 (*p* = 0.022); and higher than in the Dexa group on postoperative day 1 (*p* = 0.001; Table 6). Patients in the Mosa+Dexa group began to ambulate 20.6 ± 5.9 h after surgery, significantly sooner than patients in the Dexa group (23.4 ± 5.8 h, *p* = 0.005) or control group (23.3 ± 4.6 h, *p* = 0.008). Patients in the Mosa+Dexa group were more satisfied with their hospital experience than patients in the control group (p <  0.001) and Dexa group (p = 0.008). Length of hospitalization was shorter in the Mosa+Dexa group than in the contrl group (4.9 ± 1.4 vs 5.6 ± 1.5 d, *p* = 0.012).

**Table 6:** Other clinical outcomes

	Control (n = 80)	Dexa (n = 82)	Mosa+Dexa (n = 78)	p*	p1^†^	p2^†^	p3^†^
Time to first defecation (h)	60.7 ± 24.6	54.7 ± 15.8	36.4 ± 18.3	< 0.001	0.136	< 0.001	< 0.001
Appetite on POD 0	2.26 ± 0.67	2.34 ± 0.65	2.55 ± 0.68	0.020	0.731	0.019	0.117
Appetite on POD 1	2.38 ± 0.62	2.41 ± 0.63	2.76 ± 0.51	< 0.001	0.905	< 0.001	0.001
Appetite on POD 2	2.63 ± 0.49	2.73 ± 0.45	2.82 ± 0.45	0.030	0.306	0.022	0.443
Time to first ambulation (h)	23.4 ± 5.8	23.3 ± 4.6	20.6 ± 5.9	0.002	0.976	0.005	0.008
Satisfaction	7.7 ± 1.1	8.7 ± 1.1	9.2 ± 0.9	< 0.001	< 0.001	< 0.001	0.008
Length of hospitalization (d)	5.6 ± 1.5	5.1 ± 1.5	4.9 ± 1.4	0.013	0.095	0.012	0.683

Data presented as mean ± standard deviation. Dexa, dexamethasone; Mosa, mosapride; POD, postoperative day

p1, Control vs Dexa; p2, Control vs. Mosa+Dexa; p3, Dexa vs. Mosa-Dexa. *p values with one-way ANOVA or Wilcoxon signed-rank test and ^**†**^p values with Tukey post hoc test

No adverse events associated with mosapride including prolonged QT syndrome were observed during the study or follow-up. A total of 5 patients (including 1 abdominal pain, 2 dry mouth, 2 insomnia) experienced side effects in control group, 5 patients (1 diarrhea, 1 dry mouth, 3 insomnia) in Dexa group and 6 (in abdominal pain, 2 dry mouth and 3 insomnia) in Mosa + Dexa group without significance (*p* = 0.908, Table 7).

**Table 7:** Adverse events and side effects

	Control (n = 80)	Dexa (n = 82)	Mosa + Dexa (n = 78)	p*
Long QT syndrome	0	0	0	NA
Diarrhea	0	1 (1.22%)	0	1.000
Abdominal pain	1 (1.25%)	0	1 (1.28%)	0.556
Dry mouth	2 (2.5%)	1 (1.22%)	2 (2.56%)	0.752
Insomnia	2 (2.5%)	3 (3.66%)	3 (3.85%)	0.908
Total	5 (6.25%)	5 (6.1%)	6 (7.69%)	0.906

Data presented as mean ± standard deviation. *Dexa* dexamethasone; *Mosa* mosapride

*p values were analyzed using Fisher’s exact test

## Discussion

PONV remains a challenge in recovery after total joint arthroplasty surgery, because it creates anxiety in patients, lowers their appetite, delays ambulation and even disturbs water and electrolyte balance. This increases risk of postoperative complications and prolongs hospitalization and consensus guidelines have been formulated for the management of PONV [7]. Fifteen years have passed since the first international recommendations for the prophylaxis and treatment of PONV [19], and the incidence of PONV has fallen from approximately 80% to 20–30% [20], which is still relatively high. Our own work suggests that at least certain patient populations may show much higher incidence: we reported, for example, 48% incidence in patients who underwent total joint arthroplasty with general anesthesia [unpublished data]. Indeed, risk of PONV appears to be 11-fold higher when surgery is performed under general anesthesia than under regional anesthesia [21]. While modification of risk factors should be taken firstly according to the recommendation of latest consensus [7], some risk factors such as gender or history of smoking and PONV were non-modifiable. Moreover, in our country, total joint arthroplasty is performed under general anesthesia routinely, because of the different medical system. On the other hand, spinal anesthesia is conductive to early ambulation and postoperative anticoagulation following enhanced total joint arthroplasty. These considerations highlight the need to improve PONV prophylaxis and treatment.

In our current study, we compared a new multimodal PONV prophylaxis protocol with a traditional anti-emetic protocol involving ondansetron and/or dexamethasone. The latter two drugs are the most commonly used perioperatively to prevent PONV. Our results indicate that addition of the oral prokinetic drug mosapride can lead to lower PONV incidence and severity, especially postoperative nausea, during the first 12 h postoperatively than the use of ondansetron alone or in combination with dexamethasone, although the difference of severe PONV rate between Dexa + Mosa group and Dexa Group was not statistically significant (*p* = 0.062). Moreover, preemptive application of oral mosapride can improve appetite, bowel function, time until ambulation and length of hospital stay.

5-HT_3_ receptor antagonists and corticosteroids are the anti-emetic drugs most commonly used perioperatively to prevent and treat PONV. These drugs act by antagonizing 5-HT_3_ receptors both peripherally on vagal afferents and centrally in the area postrema. Dexamethasone is a synthetic glucocorticoid showing higher potency, greater bioavailability, and longer acting time than the most widely used antagonist, ondansetron [22–24]. Low-dose systemic dexamethasone can show good anti-emetic efficacy [25, 26], although the mechanism remains unclear. The drug may inhibit prostaglandin synthesis and endogenous opioid release, controlling pain and reducing the need for opioid drugs, which can help prevent PONV. The half-life of dexamethasone is about 36–72 h, much longer than the 4 h of ondansetron [22, 23]. Therefore, the combination of dexamethasone and ondansetron can be particularly effective at reducing PONV incidence during the first 12–48 h after surgery (Tables 3 and 4). We found that combining dexamethasone with ondansetron led to lower incidence of PONV and higher incidence of complete response than with ondansetron alone. Similarly, previous work showed that combining dexamethasone with ramosetron further reduced postoperative emesis and pain without increasing risk of wound complications [27]. Studies also have suggested that dexamethasone can help reduce postoperative pain and inflammatory responses [11, 12, 24, 27, 28], which we did not examine here. Future work should explore the full range of benefits offered by dexamethasone.

Here we investigated additional clinical benefits of the anti-emetic mosapride, a selective 5-HT_4_ receptor agonist. Mosapride can promote the release of acetylcholine and stimulate the gastrointestinal tract to promote motility, thereby improving the gastrointestinal symptoms of patients with functional dyspepsia. Mosapride can inhibit emesis induced by selective serotonin reuptake inhibitors (SSRIs) in laboratory animals [14], probably by reversing the SSRI-induced delay in gastric emptying. We found in the present study that the preemptive use of oral mosapride can provide additional anti-emetic effects beyond dexamethasone and ondansetron in total joint arthroplasty patients. In fact, mosapride may antagonize the ability of 5-HT_3_ receptor antagonists to reduce gastrointestinal motility, which may help reduce the risk of constipation that affects many total hip and knee arthroplasty patients [29]. Indeed, this motility-inducing effect may help explain why the patients in our Mosa+Dexa group showed better postoperative appetite and shorter times to first defecation and ambulation than patients who did not receive mosapride. Although some patients in Mosa + Dexa group experienced digestive side effects such as abdominal pain or dry mouth, we could not conclude the correlation between mosapride and these side effects because of non-significant difference.

As far as we know, our study provides comprehensive evidence supporting the potential clinical benefit of prokinetic drugs following total joint arthroplasty, although the results need to be interpreted in light of the following limitations. First, patients receiving mosapride alone were not included. Therefore, we cannot make the conclusion that mosapride alone was effective at reducing PONV incidence after total joint arthroplasty. Although our results implicated no incidence of prolonged QT syndrome, more further works are needed to confirm and extend our results, especially the safety profile of mosapride, because another 5-HT_4_ agonist that antagonizes 5-HT_3_ activity and that can rapidly relieve nausea induced by SSRIs was withdrawn for cardiotoxicity [30]. Second, the sample number was calculated according to our primary outcome of PONV incidence. As a result, the sample may not have been adequately powered to detect inter-group differences in secondary outcomes, such as rate of severe PONV, and complication rates. Nevertheless, previous work from our group and others suggests that perioperative dexamethasone does not increase the incidence of postoperative infection in total hip and knee arthroplasty [11, 12, 31]. Third, all participants in our study were Chinese and most (67%) were women. Thus, our findings may not be widely generalizable, because female gender is a well-established risk factor for PONV [32]. Fourth, our postoperative analgesic regimen included extensive multimodal pain control drugs and modalities such as preemptive analgesic medication and periarticular injection, which may have introduced heterogeneity into our sample. However, the rest VAS pain score at postoperative 24 h and 48 h and requirement of rescue treatment for pain were comparable among the three groups (Table 5). Fifth, our study did not investigate whether the choice of PONV management regimen affected postoperative inflammatory marker levels and pain control. This should be explored in future work, since our previous study has shown that low-dose dexamethasone can relieve postoperative pain and postoperative nausea, as well as provide additional inflammatory control and improve clinical outcomes [28]. Lastly, our study excluded diabetic patients, so future work should examine whether our results can be generalized to this patient subpopulation.

## Conclusion

Prophylactic use of concurrent dexamethasone and oral mosapride can reduce overall incidence and severity of PONV, especially postoperative nausea, as well as increase complete response rates during the first 12 h after total hip and knee arthroplasty when compared with other patients. While it seemed that oral mosapride would not provide additional effect on reducing the rate of severe PONV when compared with dexamethasone alone. Preemptive oral mosapride can also improve postoperative appetite and bowel function.

## Data Availability

The datasets used and analysed during the current study are available from the corresponding author on reasonable request.

## Abbreviations

PONV: Postoperative nausea and vomiting
THA: Total hip arthroplasty
TKA: Total knee arthroplasty
ASA: American Society of Anesthesiologists
PACU: Post-anesthesia care unit
BMI: Body mass index

## References

[1] Kurtz S, Ong K, Lau E, Mowat F, Halpern M (2007). Projections of primary and revision hip and knee arthroplasty in the United States from 2005 to 2030. J Bone Joint Surg Am.

[2] Patel A, Pavlou G, Mujica-Mota RE, Toms AD (2015). The epidemiology of revision total knee and hip arthroplasty in England and Wales: a comparative analysis with projections for the United States. A study using the National Joint Registry dataset. Bone Joint J.

[3] Lin CJ, Williams BA (2011). Postoperative nausea and vomiting in ambulatory regional anesthesia. Int Anesthesiol Clin.

[4] Sansonnens J, Taffe P, Burnand B, group ADSs (2016). Higher occurrence of nausea and vomiting after total hip arthroplasty using general versus spinal anesthesia: an observational study. BMC Anesthesiol.

[5] Sculco PK, Pagnano MW (2015). Perioperative solutions for rapid recovery joint arthroplasty: get ahead and stay ahead. J Arthroplast.

[6] Kehlet H (2013). Fast-track hip and knee arthroplasty. Lancet.

[7] Gan TJ, Diemunsch P, Habib AS, Kovac A, Kranke P, Meyer TA, Watcha M, Chung F, Angus S, Apfel CC, Bergese SD, Candiotti KA, Chan MT, Davis PJ, Hooper VD, Lagoo-Deenadayalan S, Myles P, Nezat G, Philip BK, Tramer MR, Society for Ambulatory A (2014). Consensus guidelines for the management of postoperative nausea and vomiting. Anesth Analg.

[8] Kranke P, Diemunsch P (2014). The 2014 consensus guidelines for the management of postoperative nausea and vomiting: a leapfrog towards a postoperative nausea and vomiting-free hospital. Eur J Anaesthesiol.

[9] Eberhart LH, Kranke P (2016). Postoperative nausea and vomiting: is everything now solved or still more questions than answers?. Eur J Anaesthesiol.

[10] Ross-Adjie GM, Monterosso L, Bulsara M (2015). Bowel management post major joint arthroplasty: results from a randomised controlled trial. Int J Orthop Trauma Nurs.

[11] Xu B, Ma J, Huang Q, Huang ZY, Zhang SY, Pei FX (2018). Two doses of low-dose perioperative dexamethasone improve the clinical outcome after total knee arthroplasty: a randomized controlled study. Knee Surg Sports Traumatol Arthrosc.

[12] Lei YT, Xu B, Xie XW, Xie JW, Huang Q, Pei FX (2018). The efficacy and safety of two low-dose peri-operative dexamethasone on pain and recovery following total hip arthroplasty: a randomized controlled trial. Int Orthop.

[13] Dorn S, Lembo A, Cremonini F (2014). Opioid-induced bowel dysfunction: epidemiology, pathophysiology, diagnosis, and initial therapeutic approach. Am J Gastroenterol Suppl.

[14] Mine Y, Oku S, Yoshida N (2013). Anti-emetic effect of mosapride citrate hydrate, a 5-HT4 receptor agonist, on selective serotonin reuptake inhibitors (SSRIs)-induced emesis in experimental animals. J Pharmacol Sci.

[15] Ganesan P, Sharma A, Mohanti BK, Gogia A (2010). Protracted cisplatin-induced vomiting responding to mosapride. Indian J Cancer.

[16] Watcha MF, White PF (1992). Postoperative nausea and vomiting. Its etiology, treatment, and prevention. Anesthesiology.

[17] Xiang Y, Ye W, Sun N, Jin X (2016). Analgesic and sedative effects of Dezocine and midazolam during Vitrectomy. Curr Eye Res.

[18] Reibaldi M, Fallico M, Longo A, Avitabile T, Astuto M, Murabito P, Minardi C, Bonfiglio V, Boscia F, Furino C, Rejdak R, Nowomiejska K, Toro M, Cennamo G, Cillino S, Rinaldi M, Fiore T, Cagini C, Russo A. Efficacy of three different prophylactic treatments for postoperative nausea and vomiting after Vitrectomy: a randomized clinical trial. J Clin Med. 2019;8(3). 10.3390/jcm8030391. 10.3390/jcm8030391 PMC6463101 30901867

[19] Gan TJ, Meyer T, Apfel CC, Chung F, Davis PJ, Eubanks S, Kovac A, Philip BK, Sessler DI, Temo J, Tramer MR, Watcha M, Department of Anesthesiology DUMC (2003). Consensus guidelines for managing postoperative nausea and vomiting. Anesth Analg.

[20] Habib AS, Gan TJ (2012). Postoperative nausea and vomiting: then & now. Anesth Analg.

[21] Sinclair DR, Chung F, Mezei G (1999). Can postoperative nausea and vomiting be predicted?. Anesthesiology.

[22] Spies CM, Strehl C, van der Goes MC, Bijlsma JW, Buttgereit F (2011). Glucocorticoids. Best Pract Res Clin Rheumatol.

[23] Coutinho AE, Chapman KE (2011). The anti-inflammatory and immunosuppressive effects of glucocorticoids, recent developments and mechanistic insights. Mol Cell Endocrinol.

[24] D'Souza N, Swami M, Bhagwat S (2011). Comparative study of dexamethasone and ondansetron for prophylaxis of postoperative nausea and vomiting in laparoscopic gynecologic surgery. Int J Gynaecol Obstet.

[25] Lunn TH, Kehlet H (2013). Perioperative glucocorticoids in hip and knee surgery - benefit vs. harm? A review of randomized clinical trials. Acta Anaesthesiol Scand.

[26] Kardash KJ, Sarrazin F, Tessler MJ, Velly AM (2008). Single-dose dexamethasone reduces dynamic pain after total hip arthroplasty. Anesth Analg.

[27] Koh IJ, Chang CB, Lee JH, Jeon YT, Kim TK (2013). Preemptive low-dose dexamethasone reduces postoperative emesis and pain after TKA: a randomized controlled study. Clin Orthop Relat Res.

[28] Lei Y, Huang Q, Xu B, Zhang S, Cao G, Pei F (2018). Multiple low-dose dexamethasone further improves clinical outcomes following Total hip Arthroplasty. J Arthroplast.

[29] Ho J, Kuhn RJ, Smith KM (2008). Update on treatment options for constipation. Orthopedics.

[30] Bergeron R, Blier P. Cisapride for the treatment of nausea produced by selective serotonin reuptake inhibitors. Am J Psychiatry. 1994;151(7):1084–6. 10.1176/ajp.151.7.1084. 10.1176/ajp.151.7.1084 8010370

[31] Richardson AB, Bala A, Wellman SS, Attarian DE, Bolognesi MP, Grant SA (2016). Perioperative dexamethasone administration does not increase the incidence of postoperative infection in Total hip and knee Arthroplasty: a retrospective analysis. J Arthroplast.

[32] Gan TJ (2006). Risk factors for postoperative nausea and vomiting. Anesth Analg.

